# Magnetic Stimulation in the Treatment of Urgency Urinary Incontinence: A Randomized Sham-Controlled Clinical Trial

**DOI:** 10.1007/s00192-025-06491-6

**Published:** 2025-12-16

**Authors:** David Lukanović, Anja Antič, Maja Pavčnik, Matija Barbič, Miha Matjašič, Adolf Lukanović

**Affiliations:** 1 Department of Gynecology, Division of Gynecology and Obstetrics, Ljubljana University Medical Center, 1000 Ljubljana, Slovenia; 2 Department of Gynecology and Obstetrics, Faculty of Medicine, University of Ljubljana, 1000 Ljubljana, Slovenia; 3 Health Centre Celje, 3000 Celje, Slovenia; 4 Department of Education Studies, Faculty of Education, University of Ljubljana, 1000 Ljubljana, Slovenia

**Keywords:** Magnetic stimulation, Urinary incontinence, Urgency urinary incontinence, Conservative treatment

## Abstract

**Introduction and Hypothesis:**

Magnetic stimulation is a noninvasive, painless neuromodulatory therapy that has emerged as a promising conservative treatment for urinary incontinence. This study aimed to evaluate its clinical effectiveness and safety in women with urgency urinary incontinence (UUI).

**Methods:**

In this single-centre, prospective randomised controlled trial, 70 women with UUI were randomised in a 2:1 ratio to active MS or sham treatment. Participants received 12 sessions of magnetic stimulation over 6 weeks. The active group received individually adjusted stimulation intensity, while the sham group received minimal stimulation to preserve blinding. The primary outcome was change in symptom severity measured by the International Consultation on Incontinence Questionnaire–Urinary Incontinence Short Form (ICIQ-UI SF). Secondary outcomes included bladder diary parameters (urinary frequency, urgency urinary incontinence episodes, nocturia), symptom-specific quality of life (UDI-6, IIQ-7), and patient-reported improvement (PGI-I). Outcomes were assessed at baseline and 6 months post-treatment.

**Results:**

At 6 months, 40 participants in the active group and 16 in the sham group completed follow-up. The active group demonstrated significantly greater improvement in ICIQ-UI SF scores (mean change −4.05 ± 3.23) compared to sham (−1.19 ± 1.72; *p* < 0.001). Significant improvements were also observed in urgency incontinence episodes, nocturia, and quality of life measures in the active group. No serious adverse events were reported, confirming the favourable safety profile of MS.

**Conclusions:**

This study provides new controlled evidence supporting MS as an effective, noninvasive, and well-tolerated treatment for women with UUI. These findings contribute important data on mid-term efficacy but highlight the need for further high-quality RCTs with standardised protocols and longer-term follow-up.

## Introduction

Urgency urinary incontinence (UUI) is a common and distressing condition, defined by the International Continence Society as involuntary urine leakage accompanied by a sudden, strong urge to void [1]. It is often part of overactive bladder (OAB) syndrome, which includes urgency, frequency, and nocturia, with or without UUI, and without infection or other pathology [2, 3]. UUI involves complex mechanisms, including detrusor overactivity, sensory urgency, and impaired neural control [4].

First-line treatment typically includes behavioural modifications, bladder training, and pelvic floor muscle training (PFMT). Pharmacological options such as antimuscarinics and β3-agonists are effective but often limited by adverse effects, particularly in older adults. Third-line therapies include botulinum toxin, percutaneous tibial nerve stimulation (PTNS), and sacral neuromodulation (SNM), which are effective but more invasive and costly, underlining the need for accessible, noninvasive options [5].

Magnetic stimulation (MS) is a noninvasive therapeutic modality that offers a potential intermediate step in the treatment continuum and was approved by the FDA in 1998 for urinary incontinence (UI). On the basis of Faraday’s law of electromagnetic induction, MS uses time-varying magnetic fields to generate electric currents that depolarise nerve fibres, resulting in passive muscle contractions. By stimulating the sacral roots (S2–S4), MS modulates detrusor overactivity and enhances pelvic floor and urethral sphincter function. Its therapeutic targets include afferent pudendal fibres involved in reflex bladder inhibition, as well as efferent fibres that improve pelvic floor strength via the guarding reflex. Because MS affects both afferent and efferent fibres, it has therapeutic applications across different types of UI. In SUI, MS supports continence primarily through pelvic floor and urethral sphincter strengthening, while in UUI, its efficacy is mediated via neuromodulation of detrusor overactivity and central reflex inhibition [3, 6–8]. Despite its advantages, MS remains underutilised owing to limited high-quality evidence and lack of standardised protocols. The 2025 EAU guidelines do not recommend MS for UI treatment due to insufficient supporting data [9]. Our recent systematic review (SR) focused specifically on MS for female UUI, identifying only five studies—just one a randomised controlled trial (RCT). While outcomes were generally positive, methodological heterogeneity precluded strong conclusions [3]. The current evidence base remains limited, with most studies combining incontinence types, lacking blinding, or using inconsistent protocols [3, 10, 11]. Optimal treatment parameters and long-term efficacy are still unclear. High-quality RCTs focused solely on UUI, with standardised protocols and validated outcomes, are urgently needed. We chose to focus on UUI for several reasons. Although MS has shown potential benefits in both UUI and SUI, recent advances in energy-based therapies (e.g. laser, radiofrequency) have expanded conservative treatment options for SUI. In contrast, UUI remains underserved, with limited noninvasive therapies beyond bladder training, lifestyle changes, and pharmacotherapy. We therefore aimed to evaluate the efficacy of active MS compared to sham treatment in women with UUI, with a 6-month follow-up. The primary outcome (POM) was the change in symptom severity measured using the International Consultation on Incontinence Questionnaire–Urinary Incontinence Short Form (ICIQ-UI SF), the only validated incontinence questionnaire in the Slovene language. By applying standardised urodynamic diagnostics, we ensured the inclusion of a homogeneous population with objectively confirmed UUI.

## Methods

### Study Design and Participants

**Fig. 1:**
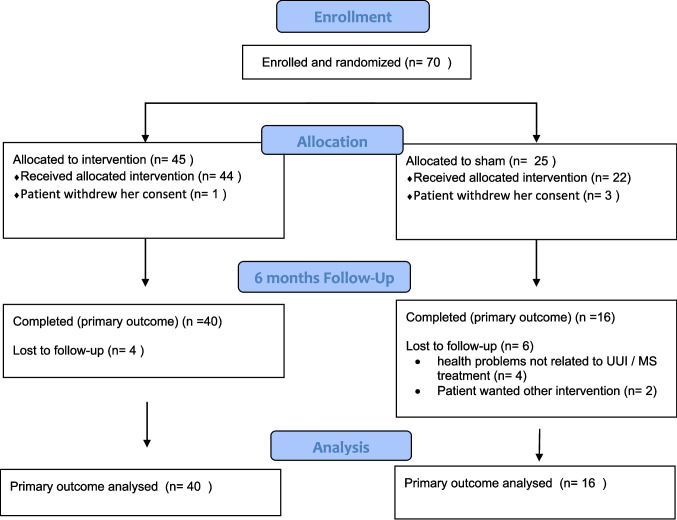
CONSORT flow diagram

This was a prospective, single-centre, sham-controlled randomised clinical trial conducted at the University Medical Centre. The recommendations of the consolidation standards of reporting trials (CONSORT) statement were followed for the reporting of this trial (Fig. 1) [12]. The trial was also registered on clinicaltrials.gov (registration number NCT05735522). Participants were recruited between Feb 2023 and January 2024 from the tertiary-level urogynaecology outpatient clinic. The diagnosis of UUI was established during the patient’s visit to our urogynaecology outpatient clinic, where a standardised initial diagnostic evaluation was performed according to the International Urogynaecology Association (IUGA)/ICS terminology for Female Pelvic Floor Dysfunctions [1], including urodynamic testing. Patients were eligible for inclusion if they were women aged ≥18 years with a clinical and urodynamic diagnosis of UUI, defined according to the ICS criteria. Symptom severity was confirmed using a 3-day bladder diary demonstrating urinary frequency ≥8 voids/day, at least one episode of UUI per day, and at least one nocturia episode per night. Additional inclusion criteria included a negative urine culture at screening and positive cough stress test at examination. Patients were excluded if they had received treatment with antimuscarinics or mirabegron within the previous 3 months. Patients with a history of prior botulinum toxin treatment, PTNS, or SNM were also excluded from the study. Additional exclusion criteria included prior pelvic floor muscle therapy—such as pelvic floor exercises, electrical stimulation, or biofeedback—during the past 3 months, urodynamic evidence of SUI (observation of urine leakage during increased intra-abdominal pressure in the absence of detrusor overactivity), pelvic malignancies, pregnancy, or pelvic organ prolapse exceeding stage II according to the POP-Q classification. Urodynamic testing was used to confirm UUI and to exclude the presence of urodynamic stress incontinence. Patients with neurological disorders affecting bladder function (e.g. multiple sclerosis, spinal cord injury) were also excluded, as well as those with any other condition that, in the opinion of the investigators, could interfere with study participation or the interpretation of results. Exclusion criteria followed the manufacturer’s safety recommendations for the Magneto STYM device (CER v7.8b) and previous publication [7]. Absolute contraindications included cardiac arrhythmia or disorders, active internal medical devices (e.g. pacemaker or medication pumps), epilepsy or suspected epilepsy, ferromagnetic or metallic implants near the stimulation site, thrombosis or thrombophlebitis, acute stages of kidney stones, and implants at the site of stimulation. Women with UUI were considered for enrolment and contacted by telephone to assess eligibility and were invited to voluntarily participate in the trial and a consent form was signed.

### Randomisation and Blinding

Participants provided written informed consent before enrolment. Randomisation was performed using a computer-generated sequence in a 2:1 ratio (intervention: sham), with allocation concealment ensured by opaque, sequentially numbered sealed envelopes. Participants were randomised in a 2:1 ratio (Active:Sham) to enhance recruitment and acceptability in a device-based study, to accrue additional safety and response-variability data in the active arm, and because this allocation entails only a minimal loss of statistical efficiency compared with a 1:1 design given the planned sample size. Randomisation was not stratified according to baseline characteristics. Participants were blinded to treatment allocation; however, owing to the nature of the intervention, treatment providers were not blinded. A total of 70 patients were enrolled and randomised between Feb 2023 and January 2024. The statistician performing the data analysis was blinded to group allocation until all analyses were completed to minimise potential bias.

### Sample Size and Power

The sample size was determined on the basis of the primary outcome, the change in ICIQ-UI SF score from baseline to 6 months. A meta-analysis of RCTs by He et al. [10] reported a pooled between-group mean difference of approximately 3 points compared with control. Accordingly, we set the target treatment effect to Δ = 3.0 points. To specify variability, we used a conservative common standard deviation (SD) of 3.5 points, consistent with the reported dispersion of ICIQ-UI SF scores in prior interventional studies [13]. Assuming a two-sided α = 0.05, 80% statistical power, and a 2:1 randomisation ratio (active:sham), the required sample sizes for a two-sample comparison of mean change (using the normal approximation to the *t*-test) were 34 participants in the active group and 17 in the sham group (total *N* = 51). To account for potential dropout and missing data—anticipated to be up to 30%, particularly in the sham arm—we aimed to randomise 70 participants to preserve at least 80% power for the primary endpoint analysis.

### Intervention Protocol

Participants received 12 therapy sessions (twice weekly for 6 weeks), with each session lasting 30 min, using a magnetic chair (Iskra Medical Magneto STYM®; Iskra Medical d.o.o., Ljubljana, Slovenia), capable of delivering magnetic field strengths up to 3 Tesla. The stimulation parameters followed the manufacturer-preset standardised program integrated into the device. Specifically, the *Urge Incontinence – Multi* program was applied (Table 1), in accordance with the device’s clinical user manual and manufacturer recommendations (Iskra Medical, CER v7.8b).

**Table 1:** Magnetic stimulation protocol parameters

Phase	Frequency	Duration	Duty cycle	Coil position
1	10 Hz	7 min	6 s on/6 s off	A + B
2	10 Hz	4 min	6 s on/6 s off	A
3	10 Hz	7 min	6 s on/6 s off	B
4	30 Hz	10 min	6 s on/6 s off	A + B
5	10 Hz	5 min	6 s on/6 s off	A + B

*A* coil under the seat; *B* coil in the lower backrest

In the active treatment group, the intensity was individually increased during each session to the highest level tolerated by the participant (range 2–100%). The stimulation intensity was individualised and adjusted according to each patient’s comfort level.

In the sham group, intensity was fixed at the minimum setting (2%) to maintain auditory and vibratory feedback while minimising neuromuscular stimulation, ensuring blinding integrity. All patients in the sham group were unblinded to their allocation at their 6-month visit and offered the active treatment therapy if they wished.

### Outcomes

Participants were assessed at baseline and 6 months post final treatment using subjective outcome measures. Participants were assessed at baseline and 6 months post final treatment using subjective outcome measures. Follow-up assessments were conducted 6 months after completion of the 12-session treatment protocol. No maintenance or booster sessions were performed during the follow-up period.

POM was the change in symptom severity, as measured ICIQ-UI SF scores between baseline and post-treatment. The ICIQ-UI SF, provides a brief and robust measure to assess the impact of symptoms of incontinence on quality of life and outcome of treatment.

Secondary outcomes included changes in bladder diary variables (voiding frequency, incontinence episodes), changes in questionaries to assess symptom burden and impact on quality of life (Urinary Distress Inventory, Short Form (UDI-6) and Incontinence Impact Questionnaire, Short Form (IIQ-7) and patient satisfaction, as assessed at 6 months using the Patients Global Impression of Improvement (PGI-I) questionnaire.

A reduction in ICIQ-UI SF, UDI-6, and IIQ-7 scores indicates a reduction in symptom severity and an improvement in quality of life.

### Statistical Analysis

Statistical analyses were performed in SPSS v28.0 (IBM Corp., Armonk, NY, USA). Baseline characteristics are summarised as mean ± SD for continuous variables and *n* (%) for categorical variables between-group baseline differences were screened with independent-samples *t*-tests. Levene’s test assessed homogeneity of variance and, when violated, we used Welch’s two-sample *t*-test.

The randomised estimate was the between-group treatment effect at 6 months. Accordingly, the primary analysis compared change from baseline between arms for continuous endpoints, reporting mean differences with 95% CIs. Because some outcomes showed deviations from normality (Kolmogorov–Smirnov and Q–Q plots), we confirmed inferences with Mann–Whitney U tests on change scores and results were concordant. As mean differences are clinically interpretable for instruments whose MCIDs are defined on the mean scale, we reported the independent-samples (or Welch’s) *t*-test results. We also reported Cohen’s *d* (0.2 small, 0.5 medium, 0.8 large).

Here should be noted that as a robustness check targeting the same estimand with potentially higher precision, we fitted ANCOVA models for each endpoint (follow-up outcome ~ treatment group + baseline value) and conclusions were unchanged.

Six-month completion proportions were compared using Fisher’s exact test (2:1 allocation; small cell counts), with risk difference (Wald 95% CI) and odds ratio (95% CI). All tests were two-sided with α = 0.05

## Results

A total of 70 women were enrolled and randomised between Feb 2023 and January 2024. Forty-five participants were allocated to the active group and 25 to the sham group. In the active group, 44 participants received the allocated intervention; one withdrew consent prior to treatment initiation. In the sham group, 22 participants received the allocated intervention, while three withdrew consent before starting treatment. At follow-up (6 months), 40/45 (89%) participants in the active arm and 16/25 (64%) in the sham arm completed the assessment. Given the small cell counts (5 and 9 non-completers) and unbalanced 2:1 allocation, we compared completion proportions using Fisher’s exact test (*p* = 0.026). The risk difference (active–sham) was 0.25 (Wald 95% CI 0.04 to 0.46) and the odds ratio for completion was 4.50 (95% CI 1.31–15.51).

These participants comprised the final analysis population. Participant flow is summarised in Fig. 1.

### Baseline Characteristics

Baseline demographic and clinical characteristics were comparable between groups (Table 2). The mean age of participants was 66.5 years, and the mean BMI was 28.45 kg/m^2^. The majority of participants were postmenopausal, and the proportions of prior gynaecological surgery, pharmacological treatment for UI, and comorbidities were similar between groups. No statistically significant differences in baseline characteristics were observed.

**Table 2:** Baseline demographic data between groups

Variable	Metric	Overall	Active	Sham
Age (years)	Mean (SD)	66.5 (9.28)	67.26 (8.76)	64.58 (10.50)
BMI (kg/m^2^)		28.45 (5,95)	28.04 (5.63)	29.47 (6.76)
Vaginal delivery (*n*)		1.71 (0.91)	1.58 (0.87)	2.06 (0.93)
Caesarean section (*n*)		0.14 (0.48)	0.20 (0.56)	0.00 (0.00)
Duration of UI symptoms (years)		7.04 (5.69)	7.63 (6.33)	5.56 (3.33)
Menopausal status	% Yes	91.1%	90.0%	93.8%
Previous gynaecological surgery		51.8%	52.5%	50.0%
Previous treatment for UI		83.9%	87.5%	75.0%
Pharmacological treatment		48.2%	50.0%	43.8%
Other		35.7%	37.5%	31.2%
MCC (mL)	Mean (SD)	302.2 (87.9)	316.9 (84.3)	274.9 (80.9)
FSF (mL)		239.2 (71.0)	243.4 (74.4)	229.2 (72.8)
FDV		256.9 (71.5)	258.6 (78.2)	248.2 (75.2)
Pdet at Qmax (cmH_2_0)		20.2 (9.2)	20.3 (8.9)	19.6 (7.5)
DO	% YES	73% (*n* = 41)	72.5 % (*n* = 29)	75 % (*n* = 12)
N		56	40	16

*BMI* body mass index, *UI* urinary incontinence, *MCC* maximum cystometric capacity, *FSF* first sensation of filling, F*DV* first desire to void, *DO* detrusor overactivity, *Pdet at Qmax* detrusor pressure at maximum capacity

Baseline urodynamic assessment demonstrated a mean maximum cystometric capacity (MCC) of 302.2 ± 87.9 mL, a first sensation of filling (FSF) of 239.2 ± 71.0 mL, and a first desire to void (FDV) of 256.9 ± 71.5 mL. The mean detrusor pressure at maximum capacity (Pdet) was 20.2 ± 9.3 cm H_2_O. Detrusor overactivity (DO) was present in 73% of participants, indicating that the majority exhibited involuntary detrusor contractions during the filling phase.

### Primary Outcome

**Fig. 2:**
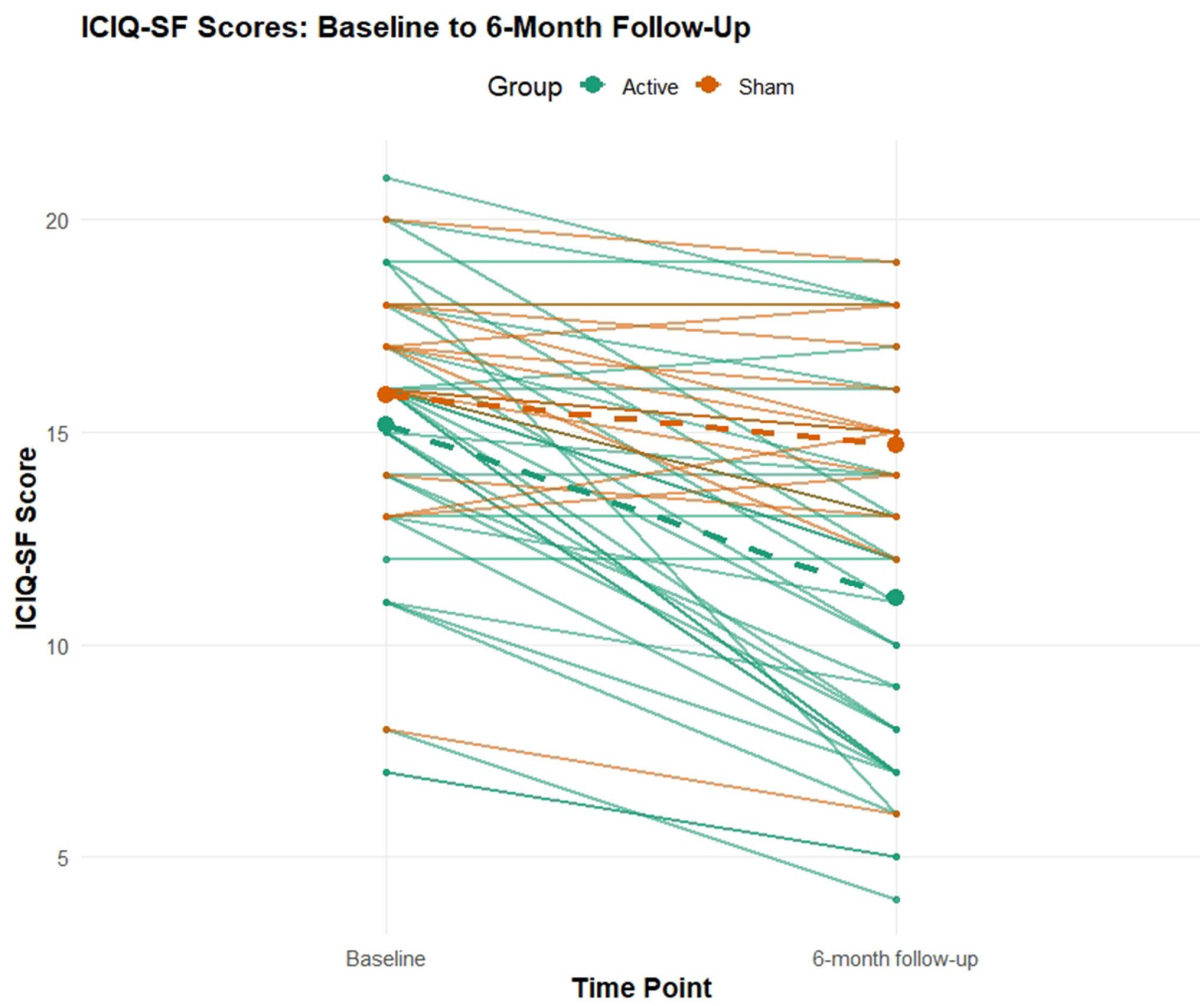
Spaghetti plot of ICIQ-SF scores at baseline and at 6-month follow-up in the active and sham groups. Solid green lines identify individual participants in the active arm, solid orange lines identify individual participants in the sham group. Dashed green and dashed orange lines present the group mean trajectories for active and sham, respectively

The ICIQ-UI SF score demonstrated a greater improvement in the active group compared to the sham group at 6 months. The mean reduction in ICIQ-UI SF score was 4.05 ± 3.23 points in the active group and 1.19 ± 1.72 points in the sham group. Levene’s test indicated unequal variances between groups (*p* = 0.005); therefore, the unequal variances *t*-test was applied. This analysis revealed a statistically significant difference in symptom improvement between the groups (t(49.32) = 4.29, *p* < 0.001, *d* = 0.99), with a mean difference of 2.86 points (95% CI 1.52 to 4.20) in favour of the active treatment (Table 3 and Fig. 2). In a sensitivity analysis using ANCOVA (follow-up ICIQ-UI SF as the outcome; predictors: treatment group and baseline ICIQ-UI SF), the effect was confirmed: adjusted mean difference −2.99 points (95% CI −4.70 to −1.28; *p* < 0.001), indicating lower (better) follow-up scores in the active arm. The group × baseline interaction (test of homogeneity of slopes) was not significant, supporting the model’s assumptions,

**Table 3:** Baseline and 6-month changes in urinary symptoms, quality-of-life measures, and patient-reported outcomes in the active and sham groups

	Active		Sham		Active	Sham		
Variable	Baseline	6 months	Baseline	6 months	Δ Mean ± SD	Δ Mean ± SD	*p* value	Cohen’s *d*
3-day incontinence episodes	6.45	2.5	4.94	4.75	+4.0 ± 3.0	+0.2 ± 2.7	<0.001	1.31
Daily urinary frequency	9.45	7.73	10.06	9.31	+1.73 ± 1.57	+0.75 ± 1.91	0.054	0.58
Nocturia	2.28	1.18	2.19	1.81	+1.10 ± 0.90	+0.38 ± 0.81	0.006	0.83
ICIQ-SF	15.15	11.1	15.88	14.69	+4.05 ± 3.23	+1.19 ± 1.72	0.001	0.99
UDI-6	56.42	40.4	43.61	42.96	+16.0 ± 14.7	+0.7 ± 8.4	<0.001	1.16
IIQ-7	63.78	46.1	67.98	64.49	+17.7 ± 18.0	+3.5 ± 11.4	0.005	0.86
PGI-I		2.05		3.88				

Values are presented as means unless otherwise indicated. Δ denotes the mean change from baseline ± standard deviation (SD). *p* values represent between-group differences in change from baseline. Cohen’s *d* indicates effect size.

*ICIQ-SF* International Consultation on Incontinence Questionnaire Short Form (0–21; higher scores indicate worse symptoms); *UDI-6* Urogenital Distress Inventory Short Form (0–100; higher scores indicate greater symptom distress); *IIQ-7* Incontinence Impact Questionnaire Short Form (0–100; higher scores indicate greater impact on quality of life); *PGI-I* Patient Global Impression of Improvement (1 = very much improved; 7 = very much worse)

### Secondary Outcomes

The bladder diaries demonstrated a greater reduction in urinary frequency in the active group compared to the sham group at 6 months. The mean reduction in daily urinary frequency was 1.73 ± 1.57 episodes in the active group and 0.75 ± 1.91 episodes in the sham group, with a borderline significant difference between groups (t(54) = 1.97, *p* = 0.054, *d* = 0.58) (Table 3).

The number of UUI episodes over the last 3 days showed a significantly greater reduction in the active group compared to the sham group. The mean reduction was 3.95 ± 2.95 episodes in the active group and 0.19 ± 2.66 episodes in the sham group. Analysis showed a statistically significant difference between groups (t(54) = 4.42, *p* < 0.001, *d* = 1.31), with a mean difference of 3.76 episodes (95% CI 2.06 to 5.47) favouring the active treatment (Table 3).

Nocturia episodes also decreased more in the active group. The mean reduction was 1.10 ± 0.90 episodes in the active group and 0.38 ± 0.81 episodes in the sham group. A statistically significant difference between groups was found (t(54) = 2.80, *p* = 0.007, *d* = 0.83), with a mean difference of 0.73 episodes (95% CI 0.21 to 1.24) in favour of the active treatment (Table 3).

The UDI-6 score improved significantly more in the active group compared to the sham group at 6 months. The mean reduction in UDI-6 score was 16.02 ± 14.73 points in the active group and 0.65 ± 8.41 points in the sham group. The analysis revealed a statistically significant difference in symptom improvement between groups (t(54) = 3.91, *p* < 0.001, *d* = 1.16), with a mean difference of 15.37 points (95% CI 7.49 to 23.24) in favour of the active treatment (Table 4).

The IIQ-7 score also demonstrated greater improvement in the active group. The mean reduction was 17.67 ± 18.00 points in the active group and 3.49 ± 11.37 points in the sham group, with a statistically significant difference between groups (t(54) = 2.92, *p* = 0.005, *d* = 0.83), with a mean difference of 14.18 points (95% CI 4.44 to 23.93) in favour of the active treatment (Table 3).

**Fig. 3:**
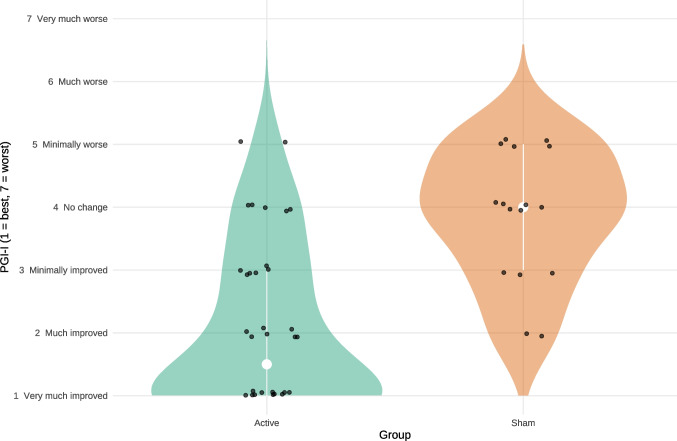
PGI-I scores by treatment group. Violin plots illustrate the distribution of PGI-I scores for the active and sham groups. Black dots represent individual patient scores. The white dot denotes the median, the thick white bar indicates the interquartile range, and the thin white line represents the 95% confidence interval

Regarding the PGI-I, patients in the active group reported better global improvement at 6 months. The mean PGI-I score was 2.05 in the active group and 3.88 in the sham group, indicating a better subjective improvement in the active treatment group (Table 3). In the violin plot of Fig. 3 the active group shows a clear concentration of responses toward lower PGI-I values (1–3), indicating that most participants reported being *much* or *very much improved*. In contrast, the sham group displays a broader and higher distribution centred around scores of 4–6, reflecting *minimal change or worsening* of symptoms. The *t*-test result show a large, statistically significant difference (*p* < 0.001 *d* = 1.5).

A sensitivity ANCOVA (follow-up outcome as the dependent variable; predictors: treatment group and the corresponding baseline value) corroborated all secondary endpoints. Adjusted mean differences (active–sham; negative values favour active because lower scores indicate improvement) were as follows: last 3-day UUI episodes −3.05 (*p* < 0.001), daily urinary frequency −1.12 (*p* = 0.019), nocturia −0.70 (*p* = 0.006), UDI-6 −12.57 (*p* = 0.004), and IIQ-7 −14.46 (*p* = 0.005). Baseline values were strong predictors of follow-up in all models, and inferences were unchanged from the primary change-score analyses. The group × baseline interaction was nonsignificant for daily urinary frequency, nocturia, UDI-6, and IIQ-7. For last 3-day UUI episodes, the interaction was small (partial η^2^ = 0.08) and did not alter the conclusion that active MS outperformed sham.

## Discussion

To our knowledge, apart from the study by Yamanishi et al. [14], this is the second RCT evaluating the role of MS in the treatment of UUI. MS has the advantages of being noninvasive, safe, and simple; it can directly treat the site of injury, pain, and/or dysfunction; and has the ability of nerve stimulation without eliciting pain, which can be a bothersome side effect of electrical stimulation [15–17]. On the basis of our analysis, we conclude that women treated with MS achieved significantly greater improvement after 6 months compared to those in the sham group. The mean ICIQ-UI SF score improved from 15.15 at baseline to 11.10 at 6 months in the active group, corresponding to a mean change of 4.05 ± 3.23 points. This exceeds the minimum clinically important difference (MCID) of 4 points for the ICIQ-UI SF, as reported by Lim et al. [13] for women undergoing nonsurgical interventions for stress urinary incontinence (SUI). The sham group showed a mean change of 1.19 ± 1.72 points, which is below the MCID threshold. Moreover, Fig. 2 illustrates that the treatment with MS produced a pronounced and consistent reduction in ICIQ-UI SF scores, whereas the sham group showed only a modest decline. The between-group difference is highly unlikely to be due to chance (*p* < 0.001), and the effect size (Cohen’s *d* = 0.99) falls within the “large” range, indicating both statistical significance and clinical relevance. Although individual responses within the MS treatment varied—with some women showing dramatic benefit and others more modest gains—most green trajectories slope downward, in contrast to the flatter and more scattered orange lines observed in the sham group.

Supporting our findings, recent meta-analyses have confirmed the efficacy of MS for UI [10, 11]. He et al. [10] analysed 11 RCTs and reported a significant reduction in ICIQ-UI SF scores (mean difference −3.03 points; 95% CI −3.27 to −2.79), decreased urinary frequency (mean difference −1.42 episodes/day; 95% CI −2.15 to −0.69), and substantial improvement in quality of life (SMD −1.00; 95% CI −1.24 to −0.76). Our results align closely with these outcomes, particularly in symptom reduction and quality of life improvements. More recently, Yang et al. [11] expanded this evidence by analysing 24 RCTs in patients with pelvic floor dysfunction. Their meta-analysis confirmed significant improvements in UI symptoms as measured by the ICIQ-SF (SMD −0.73; 95% CI −1.05 to −0.41) and in quality of life scores (SMD −0.43; 95% CI −0.82 to −0.04). However, they found no significant benefit in broader pelvic floor dysfunctions such as overactive bladder or chronic pelvic pain, suggesting that the primary benefit of MS lies specifically in symptom control and quality of life improvement for UI. In our own recent SR [3], we focused exclusively on female UUI, aiming to clarify the clinical utility of MS in this specific population. Unlike previous reviews that combined various types of UI, we analysed five studies, of which only one was a randomised, sham-controlled trial (Yamanishi et al. [14]), with the remainder being prospective cohort studies. Despite methodological differences, all studies consistently reported significant symptom improvements with MS. Our SR confirmed that MS reduces UUI episode frequency and improves patient-reported outcomes, such as the ICIQ-UI SF and quality of life scores. These results closely match our current trial findings. However, our SR also highlighted key limitations in the literature, including small sample sizes, lack of standardised stimulation protocols (regarding frequency, intensity, and number of sessions), inconsistent use of validated diagnostic tools, and limited long-term follow-up. We emphasised the need for standardised diagnostic criteria (aligned with EAU guidelines [9]), uniform treatment protocols, and consistent outcome measures to enable meaningful comparisons across studies. Although the short-term efficacy of MS was well supported, our SR identified a lack of data on long-term effects. Only one study reported follow-up beyond 12 months, showing some decline in treatment effect over time. Our present study, with 6-month follow-up, provides additional evidence on the short- to mid-term durability of treatment effects but does not address the gap in long-term outcomes. Current evidence indicates that there is limited data on long-term maintenance dosing protocols for MS in UI. Most published studies, including SRs and meta-analyses, have assessed outcomes up to 6–12 months, with significant heterogeneity in treatment regimens and follow-up durations, and no standardised maintenance schedules established in the literature. However, some reports suggest that periodic booster sessions may help sustain the therapeutic effect over time [3, 10, 11].

When comparing our results directly with Yamanishi et al. [14], both studies demonstrated significant improvements in UUI with MS. Yamanishi et al. [14] reported a reduction of 13.1 leaks/week in the active group versus 8.7 in sham (*p* = 0.038), which is comparable to our greater reduction in 3-day incontinence episodes. Both studies observed improvements in urinary frequency, though the change was only borderline significant in our trial and not significant in Yamanishi’s [14]. Notably, we found a significant reduction in nocturia, which Yamanishi et al. did not report separately. Improvements in quality of life were comparable: Yamanishi et al. [14] documented significant improvement in the IPSS-QOL score, while our study demonstrated greater improvements in the UDI-6, IIQ-7, and ICIQ-UI SF scores. Furthermore, patient-reported outcomes favoured active treatment in both trials; in our study, this was reflected by better PGI-I scores, indicating meaningful symptom relief and greater patient satisfaction. Our results also build upon our previous prospective, nonrandomised study [18], which evaluated MS in women with all types of UI, also UUI. In that cohort, patients reported significant improvements in incontinence episode frequency, ICIQ-UI SF, and quality of life scores after 10 sessions of MS, with effects sustained at 3 months. However, the absence of a sham control group and the shorter follow-up period limited the conclusions regarding treatment durability and placebo effects. In contrast, the present RCT confirms these benefits in a controlled setting and extends the evidence by demonstrating sustained symptom improvement at 6 months, thereby providing more robust support for the clinical effectiveness of MS.

Recently, Bele et al. [15] conducted an RCT evaluating MS as an adjunct to mirabegron in women with overactive bladder (OAB), mostly with UUI. They observed a significant additional reduction in weekly incontinence episodes in the active group compared to sham (43.7% vs. 24.2% reduction, *p* = 0.013) and greater improvement in IIQ-7 scores, which is consistent with our findings of improved incontinence episode frequency and quality of life. Furthermore, while Bele et al. [15] reported only short-term effects at 8 weeks, our study provides evidence of sustained improvement over 6 months, contributing important data on treatment durability.

While our study demonstrated significant improvements in UUI episodes and quality of life measures, some secondary outcomes, such as urinary frequency, showed only borderline statistical significance. This partial response may reflect the underlying mechanisms of MS, which predominantly target neuromodulation of the sacral nerve roots and pudendal nerve, pathways more directly involved in controlling detrusor overactivity and urethral sphincter function than in modulating bladder sensory afferents that influence urinary frequency. The pelvic and pudendal nerves carry both motor and sensory fibres, contributing to bladder control through modulation of afferent signaling and detrusor inhibition [19]. This interpretation is supported by Yang et al. [11], who found that MS significantly improved incontinence-related outcomes but had limited effects on broader pelvic floor dysfunctions such as dry OAB or chronic pelvic pain. It is plausible that MS more effectively restores continence mechanisms through pelvic floor activation and inhibition of detrusor overactivity, whereas its impact on sensory urgency and habitual voiding behavior is less pronounced. However, although nocturia episodes decreased significantly in the active group compared with sham, this secondary outcome should be interpreted with caution, as the study was not powered to detect differences beyond the primary endpoint. Nocturia is largely sensory-driven, typically resulting from heightened bladder afferent signaling that triggers sleep disruption. The observed improvement may therefore suggest a sensory-modulating effect of MS, consistent with its neuromodulatory action on sacral and pudendal afferents. This potential mechanism warrants further confirmation.

Regarding safety, both our study and prior research have confirmed that MS is a well-tolerated intervention [8, 10, 11, 14, 15, 18]. Similarly, our participants did not experience significant side effects, supporting the favourable safety profile of MS. These findings are consistent with our SR [7], which included 12 clinical studies and found that MS was consistently reported as a noninvasive, painless, and safe therapy for urinary incontinence, with almost no treatment-related adverse effects documented across studies. This further confirms the excellent tolerability of magnetic stimulation, making it an attractive conservative treatment option for women with UI.

Nevertheless, this study has several limitations. First, despite being one of the few RCTs, the sample size was modest (*n* = 56 at follow-up), particularly in the sham group, where the dropout rate was relatively high (36%). This may have reduced the statistical power for some secondary outcomes and limits the generalisability of the results.

Second, the trial was single-centre and the treatment sessions were not double-blinded. While participants were blinded to group allocation, the therapist delivering the intervention was aware of the treatment type, introducing the potential for performance bias. Third, the follow-up period was limited to 6 months. Although this timeframe provides insight into short- and mid-term effects, it remains unknown whether the observed improvements are sustained in the longer term. Future studies with extended follow-up are needed to assess the durability of the treatment effects. Fourth, although we applied intention-to-treat principles and conducted appropriate statistical corrections, the Kolmogorov–Smirnov test indicated deviations from normality in some variables. Nevertheless, we chose to retain the *t*-test as the primary analysis given the sample size, the robustness of Welch’s correction, and the agreement with nonparametric results. However, we reported Cohen’s *d* alongside the *t*-test results. Future studies with larger samples could confirm these findings with more robust statistical power. Finally, a limitation of this study is also the differential attrition observed between treatment groups. At 6 months follow-up, 89% of participants in the active arm and 64% in the sham arm completed follow-up. Although no discontinuations were related to adverse events, non-completion was more frequent in the sham group, primarily due to loss to follow-up and perceived lack of benefit. This imbalance may introduce attrition bias in between-group comparisons, particularly if the missing data deviate from missing-at-random (MAR) assumptions. Furthermore, the higher non-completion rate in the sham group could reduce the precision of effect estimates and limit generalisability. These factors should be considered when interpreting the observed differences in outcomes.

It should also be noted that MS has not been investigated within the context of energy-based devices (EBDs), which are currently gaining popularity and are widely studied as potential treatment options for UI. In our recent SR and meta-analysis [20], we evaluated EBD modalities such as lasers (CO_2_ and Er:YAG) and radiofrequency, demonstrating promising results for the treatment of SUI. However, our meta-analysis, which focused exclusively on high-quality RCTs, did not include MS within the EBD group. This highlights a gap in comparative research. While MS is sometimes discussed alongside EBDs in the context of noninvasive therapies, its mechanism of action is fundamentally different. Future studies should investigate how MS compares with other EBDs treatments and whether it should be categorised within the broader group of EBD therapies or regarded as a distinct neuromodulatory approach.

## Conclusion

In conclusion, MS is a noninvasive, effective, and well-tolerated treatment UUI. Our RCT contributes new evidence supporting its clinical benefit, demonstrating significant improvements in both symptom severity and quality of life at 6 months follow-up. Despite these findings, further high-quality RCTs with standardised protocols, larger sample sizes, and longer follow-up are required to confirm long-term efficacy and to establish its place among conservative treatment options for UUI.

## Data Availability

The data that support the findings of this study are available on request from the corresponding author (BOR). The data are not publicly available due to restrictions. The data contain information that could compromise the privacy of research participants.
